# To the Editors of the Medical and Physical Journal

**Published:** 1799-07

**Authors:** Thomas Peck

**Affiliations:** Higham Ferrers


### *To the Editors of the Medical and Phyfical Journal.*

GENTLEMEN,

IN the third Number of your very interefting Journal, is a propofal for a new method of removing *fleatomatous tumours*, by Profeffor WIMMER; who, in my opinion, is defervedly entitled to commendation for his ingenious treatment of them.

Whatever tends to diminifh the *hazard*, or even the *trouble*, of an operation, ought certainly to be ftrictly confulted by the furgeon; and as tumours of this nature are not *unfrequently* fo fituated, as to be extirpated by the knife with *fome degree* of danger, any other mode of removing them is certainly defirable.

I hope the following cafe may not be deemed unworthy a page in your Journal, as it may probably be in fome meafure inftrumental to the furtherance of that method of treatment, which I conceive will be found fuccefsful in moft cafes of fimilar nature.

About five weeks fince I was defired to examine a tumour, which had been eighteen years fituated on the abdomen, immediately over the left rectus mufcle, at its *half interfection* below the umbilicus.

It palpably contained fat, and appeared to weigh rather more than a pound. The man wifhed for its extirpation by the knife; but, after perufing your Journal, I thought it a fair cafe for a feton. I accordingly paffed one through its middle, and in the courfe of three days confiderable inflammation enfued, terminating in the fphacelous floughing of the whole tumour,—fo that in twelve days from the introduction of the feton, nothing remained but a trifling induration, which readily yielded to the *ung. hydrarg.*

I am very far from expecting this cafe to be a fufficient warrant for the adoption of a fimilar treatment in all tumours of fteatomatous nature; nor do I think the knife ought to be withheld in thofe inftances where it can be ufed with impunity. Still, however, I am inclined to believe, that the treatment by feton will very frequently fucceed; and whereever the ftate of the patient's ftrength is fufficient to undergo the procefs of *fphacelous feparation*, it ought frequently to be practifed.

I am, Gentlemen, very refpectfully,

Your obedient fervant,

HIGHAM FERRERS, THOMAS PECK.

*June* 12, 1799.

